# Driver Behavior Profiling and Recognition Using Deep-Learning Methods: In Accordance with Traffic Regulations and Experts Guidelines

**DOI:** 10.3390/ijerph19031470

**Published:** 2022-01-27

**Authors:** Ward Ahmed Al-Hussein, Lip Yee Por, Miss Laiha Mat Kiah, Bilal Bahaa Zaidan

**Affiliations:** 1 Department of Computer System and Technology, Faculty of Computer Science and Information Technology, University of Malaya, Kuala Lumpur 50603, Malaysia; wva180034@siswa.um.edu.my (W.A.A.-H.); misslaiha@um.edu.my (M.L.M.K.); 2 Department of Computing, Faculty of Arts, Universiti Pendidikan Sultan Idris, Tanjong Malim 35900, Perak, Malaysia; bilalbahaa@fskik.upsi.edu.my

**Keywords:** driving behavior, driver behavior profiling, driver performance, deep learning, recognition systems, aggressive driving, naturalistic driving

## Abstract

The process of collecting driving data and using a computational model to generate a safety score for the driver is known as driver behavior profiling. Existing driver profiles attempt to categorize drivers as either safe or aggressive, which some experts say is not practical. This is due to the “safe/aggressive” categorization being a state that describes a driver’s conduct at a specific point in time rather than a continuous state or a human trait. Furthermore, due to the disparity in traffic laws and regulations between countries, what is considered aggressive behavior in one place may differ from what is considered aggressive behavior in another. As a result, adopting existing profiles is not ideal. The authors provide a unique approach to driver behavior profiling based on timeframe data segmentation. The profiling procedure consists of two main parts: row labeling and segment labeling. Row labeling assigns a safety score to each second of driving data based on criteria developed with the help of Malaysian traffic safety experts. Then, rows are accumulated to form timeframe segments. In segment labeling, generated timeframe segments are assigned a safety score using a set of criteria. The score assigned to the generated timeframe segment reflects the driver’s behavior during that time period. Following that, the study adopts three deep-learning-based algorithms, namely, Deep Neural Network (DNN), Recurrent Neural Network (RNN), and Convolutional Neural Network (CNN), to classify recorded driving data according to the established profiling procedure, and selects the most suitable one for a proposed recognition system. Various techniques were used to prevent the classification algorithms from overfitting. Using gathered naturalistic data, the validity of the modulated algorithms was assessed on various timeframe segments ranging from 1 to 10 s. Results showed that the CNN, which achieved an accuracy of 96.1%, outperformed the other two classification algorithms and was therefore recommended for the recognition system. In addition, recommendations were outlined on how the recognition system would assist in improving traffic safety.

## 1. Introduction

Driver profiling is a procedure that categorizes drivers according to their behavior [1]. Driver profiling categorizes drivers’ behavior as safe or aggressive based on their actions to ensure the safety of other road users and adherence to traffic laws. Driver profiling consists of two main phases: data collection and analysis.

Data collection methods include surveys, questionnaires, simulations, roadside camera observations, and naturalistic experiments. Surveys and questionnaires may not be ideal because they rely on self-reported values [2,3,4,5,6,7,8,9,10], and the questions asked are often subjective [4,5,6,7,8,10,11,12]. A driving simulator provides a safe driving environment for participants. As a result of this, participants become more aggressive and risk-taking [5]. On-road observations, such as mounting cameras on traffic lights or tall buildings to monitor passing vehicles’ behavior, cannot be generalized due to the lack of randomization in site selection. In addition, data from cameras is imprecise, therefore, reducing the reliability of observational studies [5,8]. As a result, naturalistic experiments have become an important and reliable source of data [10,13].

In the analysis phase, the recorded driving data are categorized into labels such as “safe” or “aggressive” by either allowing drivers to report on their dangerous driving or by allowing experts to categorize the collected data as aggressive or safe. The latter is preferred due to the experts’ knowledge of the traffic regulations in the country where the experiments were conducted. Furthermore, studies comparing self-labeling to third-party labeling found that drivers think their driving performance is better than what it actually is [6].

In previous research [13], the authors developed a data acquisition system to gather naturalistic driving data from 30 participants. The study compiled a dataset of over 750 km of continuous driving data. The study also investigated the influence of factors such as gender, age, driving day, and cultural background on driving in Malaysia. This study aims to continue the work of previous research by providing a standardized model for driver behavior profiling in Malaysia based on timeframe data segmentation.

## 2. Related Work

According to the literature, there are numerous methods for collecting driving data. Since survey and questionnaire results are dependent on self-reported values, they are not appropriate for profiling [2,3,4,5,6,7,8,9,10]. The answers given by drivers are largely subjective, as drivers tend to overestimate their own performance [6]. Throughout the literature, the usage of simulators has been cited as a drawback [10,14,15,16,17]. Simulation flaws include providing a safe environment for drivers, which causes them to become aggressive [5]. Other concerns with simulations include physical restrictions and realism, simulator sickness, validity, and data accuracy [10]. Since the camera is limited to gathering data from a single spot, the findings from using on-road observations, such as placing a camera somewhere on a side road to record passing vehicles, cannot be generalized. Furthermore, because data are collected from a camera, researchers must rely on their own judgment to recognize events (such as unsafe acceleration), which is inherently imprecise [5,8,10]. The use of in-vehicle sensors to record data from naturalistic experiments is recommended as the most reliable method for data gathering [13,18,19]. As technology advances, naturalistic experiments have emerged, allowing for the capture, storage, and analysis of ever-increasing amounts of data via sensors [20]. During the experiments, the researchers must not influence the behavior of the drivers, which means that they must drive without any special instructions or interventions, ensuring that the data acquired is trustworthy for analysis. Previous studies in Malaysia aimed to collect data based on questionnaires, simulations, and observations. Table 1 lists those studies.

In the screened literature, one study in Malaysia [31] collected data utilizing in-vehicle sensors, but the experiments were not conducted in a naturalistic manner. For instance, two cones were placed on the street by the researchers, and they urged drivers to steer through the cones and not to brake, which caused the experiments to be non-naturalistic because it changed the drivers’ usual driving behavior.

After data collection, recorded data are analyzed and labeled into various levels of aggressiveness. Data labeling is usually accomplished by either allowing the drivers to report on their behavior [2] or by experts [39]. Driving profiles are established through this labeling process.

In previous publications outside of Malaysia, experts were consulted to identify aggressive behaviors. Japan’s risk consultants assigned scores to aggressive behaviors in [40]. These scores were used to create driver profiles (from 1 to 5, with 1 being the least aggressive and 5 being the most aggressive). Moreover, based on Turkish legal authority reports, researchers classified driving risk based on possible collision damage into three categories: low, medium, and high risk [41]. In addition, in another study in Turkey, risky driving behaviors and associated risk levels were assessed by Turkish traffic officers. The risky behaviors were rated from 1 to 10, and the results were used to build a Fuzzy Logic-based risk assessment model [42]. Moreover, researchers developed a framework to identify potentially aggressive driving behaviors and provided drivers with feedback that guided them towards adopting safer behaviors based on Korean Roadway Operation Guidelines. Drivers were categorized into three levels of aggressive behavior (low, medium, and high) [12]. Furthermore, researchers in [39] used inertial signals to identify safe and aggressive driving styles. Experts in Lithuania helped in identifying those safe and aggressive signals and, as a result, categorizing the drivers into two groups (aggressive and safe). Finally, researchers in [43] aimed to detect risky lane-changes during maneuvers. Drivers’ lane-changing performance was scored by evaluators. Each lane-change was given a score from one (safest) to five (riskiest). The lane changes whose degrees of risk were in the top 5% among all lane changes were classified as “risky”, while the rest were classified as “normal”.

The aforementioned established profiles cannot be applied to drivers in Malaysia because what experts consider aggressive behavior in one country differs from what experts consider aggressive behavior in another. For instance, obtaining driving data from drivers in Kuala Lumpur and labeling these data as safe or aggressive, according to publications from the United States, presents numerous challenges due to the disparity in traffic laws and regulations between the two countries. This is why experts’ views on such topics are valuable. The fact that the articles in the screened literature are yet to incorporate the viewpoint of experts from MIROS (Malaysian Institute of Road Safety Research) on how to establish driver profiles based on the country’s traffic laws and regulations makes their applicability in Malaysia unreliable at best. Another problem with existing driver profiles is that it is impractical to categorize drivers as aggressive or safe since their behavior changes over time while driving.

Previous researchers designed recognition systems that use machine-learning algorithms to parse driving data, learn from that data, and then classify the data based on what they learn. The reason for using machine-learning approaches rather than traditional approaches, such as rule-based methods, is that machine-learning is an alternative approach that can help address some of the issues with traditional methods. For example, the expert may examine several driving scenarios and determine which are aggressive and which are not. It is not important to the algorithm how the expert arrived at his decision, only what his decision was. The use of deep-learning algorithms for categorization issues is becoming more popular these days, thanks to the rising capabilities of modern computers’ GPUs. Developing a driver behavior recognition system that adopts existing deep-learning algorithms such as CNN, DNN, and RNN has been recommended in the literature [10].

It’s worth mentioning that the majority of the studies in the screened literature were focused on acquiring driving data and statistically assessing those data. Few of them developed recognition systems for driver profiling, and the deep-learning-based ones were quite rare. Table 2 summarizes some of the current methods in use in these systems.

The main objectives of this study are:

1. To utilize naturalistic driving data in establishing a reliable model for driver behavior profiling in Malaysia based on timeframe data segmentation. The profiling process should aid in recognizing the changes in drivers’ behavior over time.
2. To modulate three major deep-learning algorithms (namely, DNN, RNN, and CNN) into classifying driving data in accordance with the established profiles.
3. To compare the performance of the modulated models to select the most suitable one for the recognition system.

## 3. Deep-Learning Models

**Figure 1:**
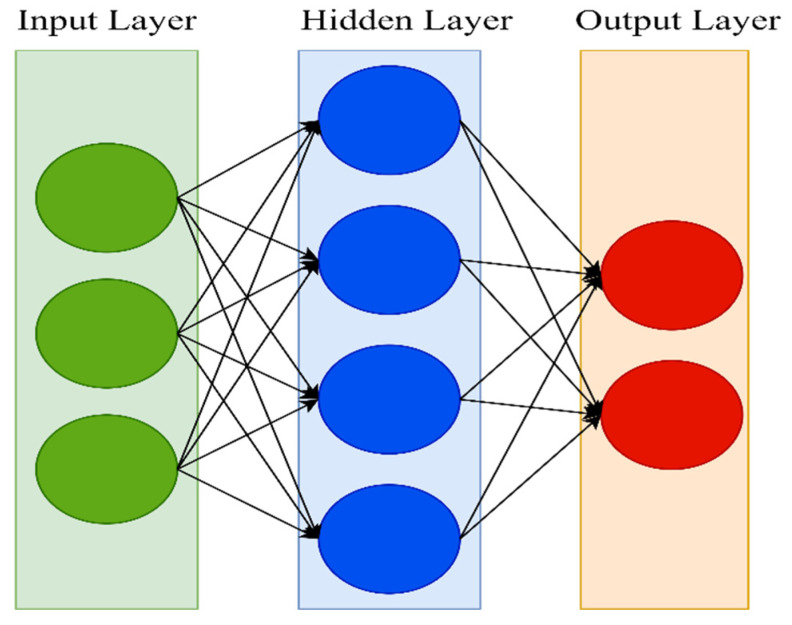
ANN architecture.

The main advantage of deep-learning over traditional methods is that the feature selection process is completely automated using a general-purpose learning procedure, with no human intervention. Deep-learning algorithms have shown outstanding performance in several fields, including speech recognition [45,46], natural language processing [47,48], computer vision [49,50], and bioinformatics [51,52], thanks to their specifiable hierarchical learning depths. Neural networks come in a variety of forms. An artificial neural network (ANN) is a type of artificial intelligence that aims to mimic the learning process that people utilize to acquire specific types of knowledge. Artificial neurons, similar to biological neurons in the brain, are present in ANN and are used to identify and store information. Neural networks are made up of layers of neurons. These neurons are the core processing units of the network. The input layer receives the input, and the output layer predicts the output. Between the input layer and the output layer, there exists a hidden layer that holds information about the relevance of an input and also makes associations between the importance of input combinations. The neurons of each layer are connected to neurons of the next layer through channels. Each of these channels is assigned a numerical value known as weight. The inputs are multiplied with the corresponding weights, and their sum is sent as input to the neurons in the hidden layer. Each of these neurons is associated with a numerical value called the bias, which is then added to the input’s sum. This value is then passed through a threshold function called the activation function. The result of the activation function determines if the particular neuron will be activated or not. An activated neuron transmits data to the neurons of the next layer over the channels. This process is called forward propagation. In the output layer, the neuron with the highest value determines the output. These values are basically probabilities. The predicted output is compared against the actual output to realize the error in prediction. This information is then transferred backward through the network in a process called backpropagation. Using this information, the weights are adjusted, and the cycle of forward propagation and backpropagation is iteratively performed with multiple inputs until the network predicts the output correctly in most cases. This brings the training process to an end. Figure 1 shows the architecture of an ANN.

**Figure 2:**
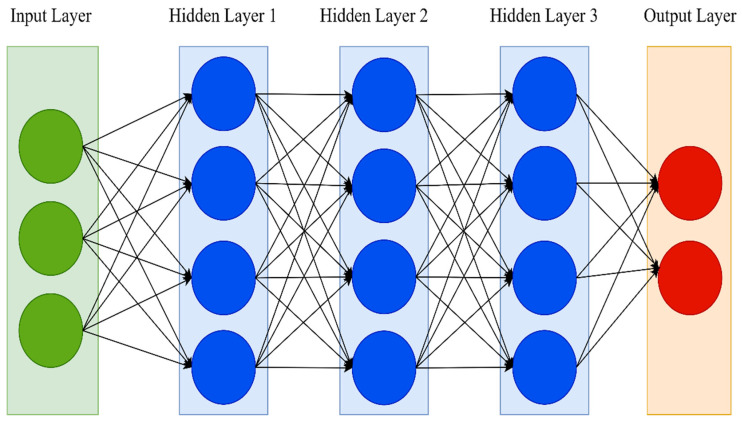
DNN architecture.

Deep neural networks (DNNs) are an extension of the traditional ANN. One hidden layer in a traditional neural network is just too shallow. DNNs, on the other hand, have many hidden layers. The number of hidden layers is the main distinction between ANN and DNN. Simple neural networks typically have only one hidden layer and may necessitate a feature selection procedure. A DNN, on the other hand, has two or more hidden layers and can perform optimal feature selection and model adjustment while learning [53]. As a result, the term “deep” refers to a model’s layers having several layers. Figure 2 shows the architecture of the DNN.

**Figure 3:**
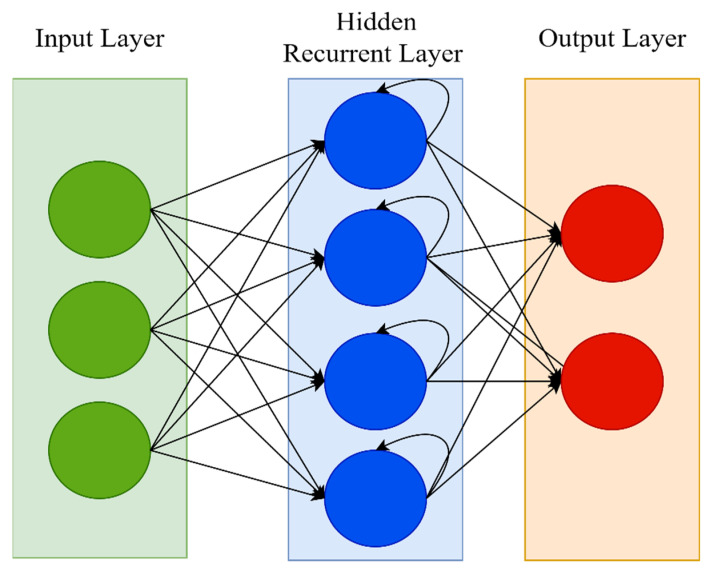
RNN architecture.

A recurrent neural network (RNN) is a type of artificial neural network that works with time series or sequential data. RNN is distinguished by its “memory,” which allows it to impact current input and output by using knowledge from previous inputs. RNNs can recall significant information about the input they receive thanks to their internal memory, allowing them to anticipate what will happen next with great accuracy. While typical DNNs presume that inputs and outputs are independent of one another, RNNs’ output is reliant on the sequence’s prior elements. This is why RNN is often preferable for applications that need sequential inputs, such as speech and language [54]. The feed-forward neural network (FNN) has a number of flaws, including its inability to handle sequential data, the fact that it only analyzes the current input, and the inability to recall previous inputs. However, owing to its internal memory, an RNN deals with sequential data, manages inputs of varying lengths, takes both current and previously received inputs, and recalls prior inputs. In some ways, RNNs are the most powerful of all neural networks; they are generic computers that outperform FNNs [53]. In RNN, the input layer receives and analyses the neural network’s input before passing it on to the middle layer. Multiple hidden layers can be found in the middle layer, each having its own activation functions, weights, and biases. The different activation functions, weights, and biases are standardized by the RNN, ensuring that each hidden layer has the same characteristics. Figure 3 shows the architecture of the RNN.

**Figure 4:**
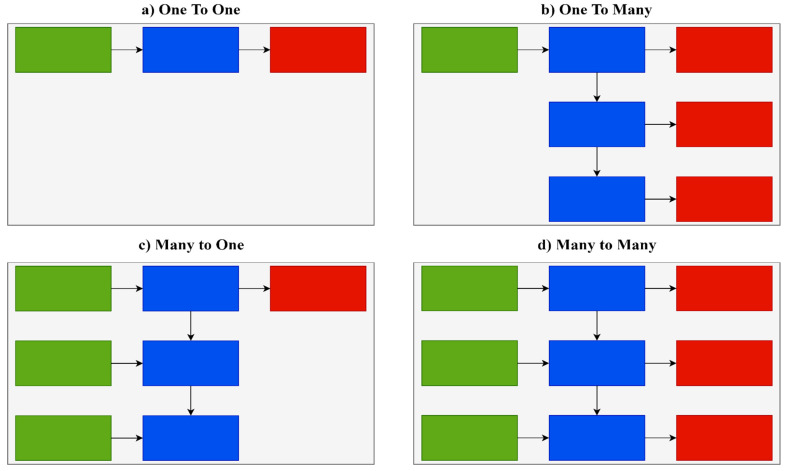
RNN types. (**a**)—one to one, (**b**)—one to many, (**c**)—many to one, (**d**)—many to many.

There are several different types of RNNs with varying architectures. One-to-one architecture is used in the majority of traditional neural networks. One-to-many architecture is implemented in situations where multiple outputs are given for a single input, such as in music or image generation. An example of the one-to-many type is predicting the caption of an image. Many-to-one architecture is implemented in situations where a single output is given for multiple inputs. These networks are often used in sentiment analysis and emotion detection. An example of the many-to-one type is classifying whether customers’ feedback is positive or negative. Many-to-many architecture is implemented in situations where multiple outputs are given for multiple inputs. It is largely used in language translation systems, such as translating sentences from English to Spanish. Figure 4 shows various RNN types.

In this research, the many-to-one type is utilized because there are many inputs for the network, such as speed, acceleration, deceleration, distance to vehicles ahead, and steering, and it is a binary classification problem, as drivers are either classified as safe or aggressive.

There are other several variations to RNN such as Bidirectional Recurrent Neural Network (BRNN) which uses inputs from future time steps to improve network accuracy. Gated Recurrent Unit (GRU) tackles the vanishing gradient problem. It has a reset and update gate that determines which information is to be retained for future predictions. Long Short-Term Memory (LSTM) is also designed to address the vanishing gradient problem. It employs three gates to help decide which information to keep: input, output, and forget.

**Figure 5:**
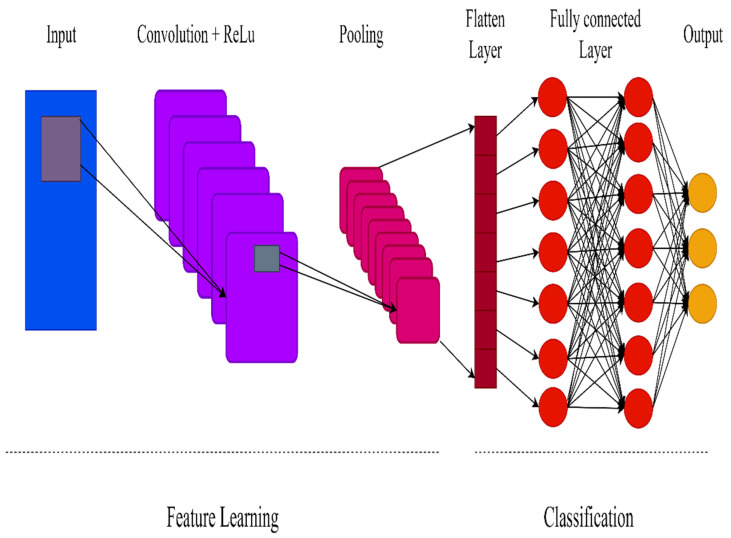
CNN architecture.

Convolutional neural networks (CNNs) are distinguished from other types of neural networks by their higher performance when inputs include images, voice, or audio signals. CNNs have replaced general matrix multiplication in standard neural networks. This reduces the number of weights in the network, thereby reducing its complexity. The CNN topology makes use of spatial relationships to decrease the number of parameters in the network, which improves performance when standard backpropagation algorithms are used. Additionally, the CNN model requires minimal preprocessing. With the rapid advancement of computation techniques, GPU-accelerated computing approaches have been used to more efficiently train CNNs. Today, CNNs have been effectively applied to various domains such as handwriting and speech recognition, face detection, image classification, and natural language processing. CNNs have three distinct layer types: convolutional, pooling, and fully connected. The first layer of the CNN is the convolutional layer. While additional convolutional layers or pooling layers can be added after the convolutional layer, the fully-connected layer is the last layer. The convolutional layer is the fundamental building block of a CNN, as it is responsible for the majority of the computation. It is composed of three components: input data, a filter, and a feature map. A CNN employs Rectified Linear Unit (ReLU) transformation to the feature map following each convolution operation, bringing nonlinearity into the model. By mapping negative values to zero and preserving positive values, the ReLU enables faster and more successful training. Pooling simplifies the output by performing nonlinear downsampling, which minimizes the number of parameters the network must learn. While some information is lost in the pooling layer, it improves the CNN by reducing complexity, increasing efficiency, and reducing the danger of overfitting. Each node in the output layer is connected directly to a node in the previous layer in the fully-connected layer. This layer performs classification using the features extracted by the previous layer and their associated filters. While convolutional and pooling layers frequently employ ReLu functions, fully connected layers frequently employ a Softmax activation function to accurately classify inputs, producing a probability between 0 and 1. Figure 5 shows the architecture of the CNN.

In [55], the authors attempted to use deep-learning in the analysis of driving behavior using GPS data. The authors studied the performance of CNNs using 1D convolution and RNNs. As a result, this technique effectively extracted high-level and interpretable information and was capable of describing complex driving patterns. Additionally, deep-learning algorithms outperformed classical methods greatly when it came to identifying the driver based on GPS driving patterns.

Detecting fatigued drivers is another application of deep-learning in driver behavior analysis [56]. The primary method in this situation was based on computer vision techniques. CNN was used to identify latent facial features and complex non-linear feature interactions. The model attained an accuracy of almost 92%. The proposed system could be used in real-time to alert drivers on their drowsiness and help them avoid traffic accidents.

In this research, deep-learning algorithms were utilized to develop a recognition system for driver profiling in Malaysia. The hyperparameters of these algorithms were fine-tuned in order to achieve optimum performance. Section 5 discusses the modulation process of the learning models.

## 4. Data

In the previous research, a total of 30 individuals were enlisted to participate. To ensure the sample was diverse enough in its gender and age representation. The participants ranged from 20 to 69 years old, with the youngest being 20 and the oldest being 69. They had an average of 22.28 years of driving experience, with a range of 2 years to 51 years in between them. The average age was 40.96 years old. In this case, 15 participants were males, and 15 were females. In addition, 15 participants were locals, and 15 were foreigners. The selected route was approximately 25 km long and ran through two cities: Kuala Lumpur and Serdang. The route consisted of various road types, such as highways, roundabouts, intersections, and tunnels. To ensure consistency and exclude the possibility that external factors such as weather and visibility would affect the data collection process, experiments were conducted during the same period (9 AM–12 PM) in clear, sunny weather. Only the participating driver was present inside the vehicle during the experiments, and his/her movements were tracked by a standalone GPS. If the driver deviated from the designated route or unexpectedly stopped during experiments, the collected data were scrapped, and the data collection process was repeated from start to finish. The experiments spanned a total of 1148.85 min, during which over 750 km of driving data were accumulated.

The data acquisition system consisted of various sensors, including an onboard diagnostics (OBDII) reader (ELM327, Elm Electronics, Canada), a lidar (LIDAR-Lite v3, Garmin, Switzerland), two ultrasonic sensors (HC-SR04, MCM, China), an inertial measurement unit (IMU) (MPU6050, TDK Corporation, United States), and a standalone global positioning sensor (GPS) (Seeworld, China). These sensors were configured to record data every second. The OBDII was utilized to detect the vehicle’s speed during experiments. Speed data were transferred to a smartphone (Samsung Galaxy S21, South Korea) via Bluetooth and saved in Excel format (Microsoft, United States). The lidar was used to capture the distance between the experimental vehicle and the vehicles in front of it. Since lidars’ accuracy usually suffers in the detection of vehicles in short distances, ultrasonic sensors are used to capture distances in close range. An FPGA (field-programmable gate array) (De-10-Nano, Terasic, Taiwan) was configured and programmed to simultaneously record distance data from the ultrasonic sensors and the lidar sensor. Distance data above four meters were obtained from the lidar, while distance data less than four meters were obtained from the ultrasonic sensors. Such settings ensured detection accuracy of no less than 97% at distances of up to 50 meters. The distance data collected by these sensors were stored in text format on the FPGA’s SD card. The IMU was installed inside the vehicle’s steering wheel to track the steering behavior of the drivers. The steering data were delivered to a Raspberry Pi (Raspberry PI 4-Model B-4GB, Raspberry PI Foundation, United Kingdom) via an antenna. These steering data were saved in text format on the Raspberry Pi’s SD card.

## 5. Methodology

**Figure 6:**
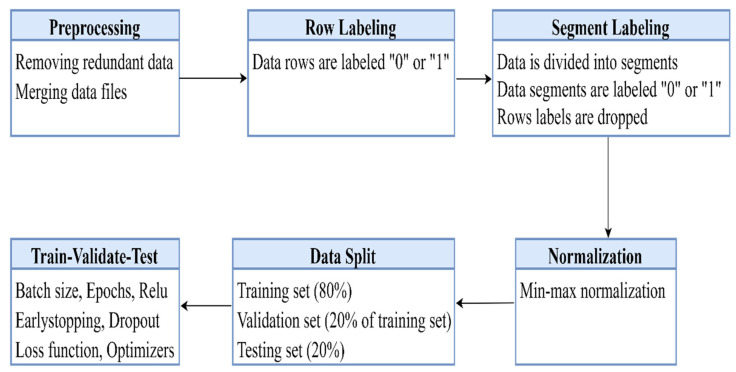
Proposed methodology.

This section discusses the processing of collected driving data, the profiling phase, and the modulation procedure of the classification algorithms, which is primarily comprised of six phases as shown in Figure 6 below.

### 5.1. Preprocessing

Data were preprocessed by deleting duplicated rows and null values. This phase reduces the dimensionality of data by removing irrelevant data. The data were preprocessed in three stages. In the first stage, irrelevant data recorded during experiments, such as engine load, fuel pressure, and kilometers traveled per liter, were removed. Such data were recorded because the OBDII reader automatically extracts that information from the vehicle’s engine. In the second stage, extra data recorded before experiments started and after experiments ended were deleted. This usually happens when the driver has not started driving yet but the acquisition system was recording data, or when the driver had stopped driving, but the acquisition system was still recording data. In the third stage, missing data, null values, blank values, and duplicated data were removed. This usually happens when there were vehicles on the road for the lidar and ultrasonic sensors to detect. Data from each driver were recorded, originally in separate files by the data acquisition system, and a merging process was implemented to work on a single file.

Experts identified speed, acceleration, deceleration, distance to vehicles ahead, and yaw steering rotation as being critical factors for identifying safe and aggressive behaviors. The data acquisition system recorded three of those five parameters, namely, speed, distance to vehicles ahead, and steering. The remaining two parameters, i.e., acceleration and deceleration, were mathematically derived and calculated from speed. Acceleration and deceleration represent the change in velocity (Δv) over the change in time (Δv), which Δv/Δt can denote. If the result was positive, it would be an acceleration. If the result was negative, it would be deceleration.

Determining when drivers are considered aggressive with regards to these five parameters was based on the Malaysian highway code, traffic regulations, published articles, and thorough discussions with experts. Table 3 shows the criteria for identifying safe and aggressive behaviors, adopted from the authors’ previous research [13].

### 5.2. Row Labeling

By now, the dataset consisted of five columns: speed, acceleration, deceleration, distance, and yaw steering. Each row represented a second-by-second snapshot of driving data collected by the data acquisition system. At any given second, if the driver failed to meet any one of the safety criteria listed in Table 3, the corresponding row was given a safety score of 1, indicating that the driver was dangerous at that moment. Otherwise, the corresponding row was given a safety score of 0. The following syntax demonstrates the row labeling process:

If ((distance to vehicle ahead > distance_limit) OR (speed > speed_limit) OR (acceleration > acceleration_limit) OR (deceleration > deceleration_limit) OR (yaw_steering > yaw_steering_limit)) {

row_label = “1”;

}

Else {

row_label = “0”;

}

Table 4 illustrates this row-labeling method. Since the driver breached the safety criteria for one or two of the parameters, the labels on rows 1, 3, 5, 6, 8, and 9 were given a safety score of 1. These offenses are colored red. Rows 2, 4, 7, and 10 were given a safety score of 0 since the driver did not breach any of the safety criteria. Safety scores with a value of 1 indicate aggressive behavior, whereas labels with a value of 0 indicate safe behavior.

It is important to note that the rows were labeled in the majority of cases during the profiling procedure using the rules specified in Table 3. However, on a few occasions, the expert disregarded the rules in favor of his judgment. For instance, when the distance between the experimental vehicle and the vehicles ahead was less than 4 m, the driver was deemed aggressive in the majority of situations. However, in a few instances, the expert disregarded this rule due to the vehicle’s extremely slow speed, which indicated that the driver was stuck in traffic, and it is common for vehicles to be closer together in traffic jams. Additionally, assuming the acceleration was 3.5002 m/s^2^, the expert determined that the driver was safe due to the fact that this fraction of 0.002 m/s^2^ is too negligible for the driver to be considered aggressive. For those rare occasions, we lack precise principles or guidelines, as labeling was made primarily based on expert judgment. Using traditional programming fails on those occasions, and learning-based algorithms are specifically designed to overcome such challenges. As a result, deep-learning-based models can attain a higher level of accuracy than traditional programming because the algorithm learns from the data and formulates its own rules based on what it has learnt.

### 5.3. Segment Labeling

Accidents, according to experts, oftentimes happen in a matter of seconds. As a result, it is prudent to create a system capable of recognizing the ever-changing behavior of drivers. Furthermore, partitioning the dataset into seconds-based segments increases the number of segments on which the classification algorithms can be trained and tested, which subsequently improves their accuracy. Let us assume the dataset has 20 h of accumulated driving time. When the dataset is broken into one-hour segments, it produces 20 segments. However, splitting the dataset into one-minute segments produces 1200 segments. Moreover, splitting the dataset into one-second segments results in 72,000 segments. Thus, partitioning the dataset into second-based segments is logical. Experts advised dividing the dataset into segments between one and 10 seconds. This is because accidents usually happen in a matter of seconds, making it critical to develop a system capable of detecting the changes in drivers’ behavior that result in accidents. Such data segmentation improves the model’s performance and provides a better understanding of driving behaviors.

After partitioning the dataset into segments, the number of aggressive and safe rows within each segment determines whether the segment is categorized as aggressive or safe. If the number of safe rows exceeded the number of aggressive rows, the segment was given a safety score of 0, indicating the driver was safe during that time period. However, the segment was given a safety score of 1 if the number of aggressive rows was equivalent to or exceeded the number of safe rows, indicating the driver was aggressive during that time period. The following syntax demonstrates the segment labeling process:

If (sum row_label = “0” > sum row_label = “1”) {

segment_label = “0”;

}

Else {

segment_label = “1”;

}

Afterward, rows’ safety scores were dropped because they were no longer required, and segments’ safety scores were kept for training the classification algorithms.

### 5.4. Data Normalization

Normalizing data in deep-learning is beneficial because features have varying scales, which can lead to poor data modeling. As a result, data are normalized to bring all features to the same scale [57,58]. The following min-max equation was used to normalize a feature’s original data so that it fell between 0.0 and 1.0:

Zi = (Xi − Min (X))/(Max (X) − Min (X))

where Zi represents the normalized value, Xi represents the original value, Min (X) represents the minimum value in the dataset, and Max (X) represents the maximum value in the dataset.

### 5.5. Data Split

Stratified sampling was used to insert segments into three types of datasets: the training set, the validation set, and the testing set. 80% of the resultant safe and aggressive segments were included in the training set. The testing set included the remaining 20% of the resultant safe and aggressive segments. Finally, the training set was subdivided into another set known as the validation set, which accounted for 20% of the training set.

### 5.6. Train-Validate-Test

#### 5.6.1. Training

The training set is the set of data used to train the model. For the training process, hyperparameters such as batch size, epochs, activation functions, learning rate, and optimizers were fine-tuned to achieve optimum performance.

Batch size is a hyperparameter that refers to the number of samples that will be passed through the network at one time. For instance, if the batch size is set to 20 during training, then 20 data are passed at a time until all the data are passed through training to complete one epoch. Large patch sizes could lead to poor generalization and degradation of accuracy [59,60,61].

Epoch is also a hyperparameter that defines the number of times the learning algorithm will work through the entire training set. Therefore, an epoch refers to one cycle through the training set. During each epoch, the model is trained repeatedly on the same data in the training set, and it will continue to learn about the features of that data to accurately predict the data in the test set. Thus, epochs play an integral part in a model’s training process as the number of epochs used helps decide whether the data is overtrained, which may lead to overfitting [62]. Overfitting occurs when the model becomes very good at classifying the data in the training set but is not as good at classifying the data that it wasn’t trained on.

In this study, through trial and error, batch size and the number of epochs were adjusted to enhance the performance of the models.

During training, the initial learning rate, a hyperparameter that controls how much to change the model in response to the estimated error each time the model weights are updated, was set to 0.001, which is considered a good starting point in optimizing neural networks [63].

An activation function is added to the neural network to help it learn complex patterns in the data. It decides whether the neuron’s input to the network is important or not by the process of prediction. ReLU is the most widely used today and is considered far more computationally efficient when compared to other functions such as the sigmoid and tanh functions [64]. Even though sigmoid is usually employed in binary classification and Softmax is usually employed in multiclass classification, employing Softmax should give the same results for binary classification because it is a generalization of sigmoid for a larger number of classes. To ensure consistency in the use of activation functions across the three deep-learning algorithms, ReLU was employed in the hidden layers, and Softmax was employed in the output layer.

#### 5.6.2. Validation

The validation set is a set of data separate from the training set that is used to validate the models during training. During the training phase, the models are training on the data in the training set while simultaneously validating the data in the validation set. One of the reasons the validation process is vital during training is to ensure the models are not overfitting to the data in the training set. Therefore, during training, if the results of the validation data are just as good as the results given for the training data, then the models are likely not overfitting. On the other hand, if the results on the training data are excellent but the results on the validation data are lagging, then the models are likely overfitting. As a result, this process was designed to help identify when overfitting starts to occur so that training can be stopped. In this study, Earlystopping function was used to prevent the training algorithm from running too long. The Earlystopping function allows one to specify an arbitrarily large number of training epochs and stop training once the model’s performance stops improving on a holdout validation dataset [65].

Another technique used in this study to prevent overfitting is Dropout (also called dilution), which is a regularization technique that reduces overfitting in neural networks by preventing complex co-adaptations on training data [66,67,68]. Dropout randomly ignores some subsets of the nodes in a given layer during training, i.e., drops out the nodes from the layer. Such a technique was used to force the neural network to learn more robust features useful in conjunction with many different random subsets of the other neurons.

The loss function (also called error function or cost function) is a crucial component of neural networks that computes the distance between the current output of the algorithm and the expected output. It is used to evaluate how the algorithm models the data as it quantifies the error between the output of the algorithm and the given target value. For binary classification problems, Binary Cross-Entropy is commonly used [69], and it predicts the probability to actual class output, which can be either 0 or 1. In this study, Binary Cross-Entropy was used for evaluating the algorithms in modeling the data.

The optimizer updates the model in response to the output of the loss function. It assists in minimizing the loss function by changing the attributes of the neural network, such as weights and learning rate, to reduce the losses. Adam (Adaptive Movement Estimation) is considered the best optimizer when training the neural network because it is too fast, converges rapidly, and rectifies the vanishing learning rate [70]. Nadam is an extension of Adam. Sometimes, using Nadam instead of Adam results in a little faster training time and better accuracy [71]. In this study, Nadam achieved better accuracy for training the DNN model. Adam achieved better accuracy for training the RNN and CNN models.

#### 5.6.3. Testing

The test set is a set of data used to test the model after being trained. This test set is separate from both the training set and the validation set. The model predicts the output of the data in the test set. The test set results were used to evaluate the performance of the three models.

## 6. Results

This section discusses the validation results and performance results of the classification algorithms.

### 6.1. Validation Results

**Figure 7:**
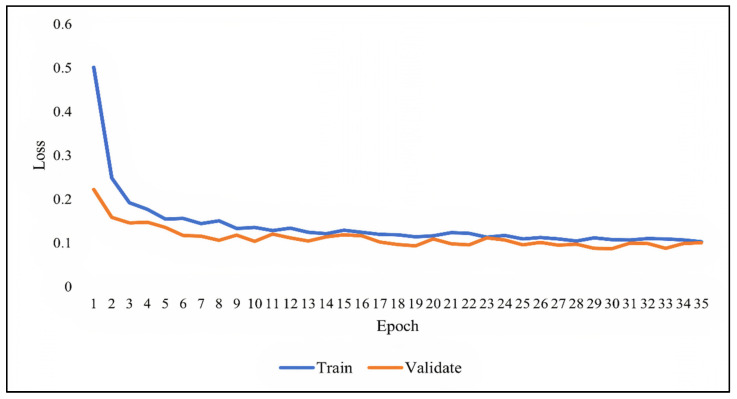
DNN learning curve during training.

**Figure 8:**
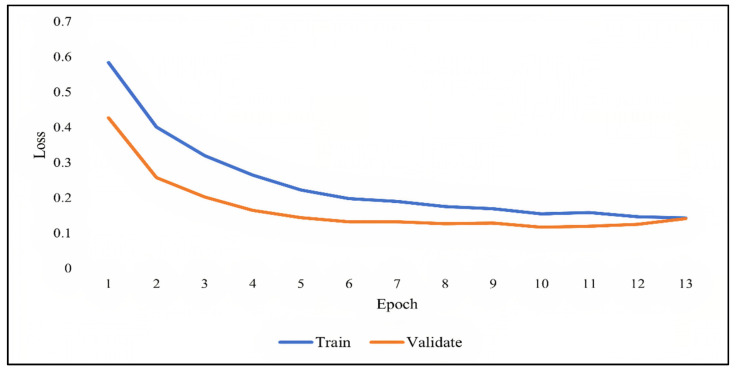
RNN learning curve during training.

**Figure 9:**
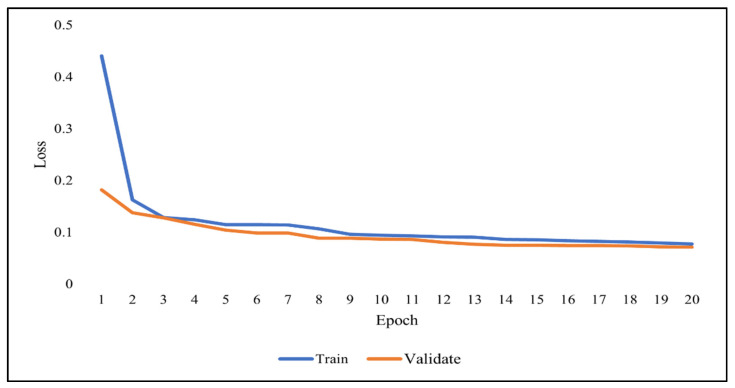
CNN learning curve during training.

As previously discussed, overfitting occurs when the model becomes very good at classifying the data in the training set but is not as good at classifying the data that it wasn’t trained on. Since the validation set is separate from the training set, it can be used to validate the models. This is achieved by comparing the learning curves of the models on both the training set and the validation set. The training learning curve is calculated from the training set and provides an understanding of how well the model is learning. The validation learning curve is calculated from the validation set and provides an understanding of how well the model is generalizing. Figure 7, Figure 8 and Figure 9 show the learning curves of DNN, RNN, and CNN algorithms, respectively, during training on both the training and validation datasets. The number of epochs is represented on the horizontal axis, while the losses (errors) are represented on the vertical axis. A general rule of thumb is that models’ mistakes are low when the learning curve is decreasing with relation to losses. Overfitting is identified when the training learning curve continues to decrease with respect to losses while the validation learning curve decreases to a certain point only to begin increasing again.

It can be observed from the figures, that the training and validation learning curves both decreased with respect to losses, to the point of stability with a minimal gap between them, indicating good-fit modeling. The training process was stopped at epochs 35, 13, and 20 for DNN, RNN, and CNN algorithms, respectively, before overfitting could begin.

### 6.2. Performance Results

A confusion matrix was deployed to rate the performance of the classification algorithms. Using a combination of true positives (TP), true negatives (TN), false positives (FP), and false negatives (FN), various metrics such as accuracy, recall, precision, and f-measures were calculated. Table 5 summarizes the main evaluation metrics reported in the article reference [10].

Accuracy is considered to be the most intuitive performance measurement, and in general, high accuracy means good modeling. This is largely true when the values of false positives and false negatives are almost the same. In cases where an imbalanced class distribution exists, researchers oftentimes look for an f-measure to evaluate the performance of their models.

The test set data were utilized to evaluate the performance of the three classification algorithms. At first, the data were partitioned into 1 s length segments, and the performance of the classification models was determined on how accurately they can predict the correct label of the segment. Then the data were merged to form 2 s length segments, and the performance of the classification models was measured again. The process was repeated until the classification models were tested on segments of 10 s length. Table 6 demonstrates the performance of the algorithms during testing on segments of 1 to 10 s in length.

The results of training accuracy and testing accuracy were very similar for each model on all segments, indicating once again that the models have no overfitting issues. The DNN and the RNN produced roughly equal performances, but the CNN provided a significantly greater performance than the other two. When the segment length was set at 2 s, CNN achieved the best accuracy (96.1%) and the best f-measure (95.2%). DNN, on the other hand, had the lowest accuracy (82.8%) and the lowest f-measure (81.5%) when the segment length was set to 9 s. Based on the results, CNN was selected for the proposed recognition system.

## 7. Discussion

Previous studies in Malaysia collected driving data using surveys/questionnaires [24,25,32,33,35,36,38], observations [21,22,23,27,28,29,30,37] and simulations [26,34]. Most of these techniques have been criticized for being biased [2,3,4,5,13,14,72]. As far as the author’s knowledge, only one study in Malaysia collected data using experiments [31]. Still, those experiments were conducted in a non-naturalistic manner. In this study, driving data were collected in naturalistic experiments. There were no instructions given to participants on how to drive to capture their naturalistic driving style, which is widely recommended throughout the literature. Since data were collected from various sensors, data preprocessing was applied in phase-1 to remove the redundant recorded data and make it suitable for training the model, therefore increasing its accuracy and efficiency.

Most of the profiling processes in the studies mentioned above were based on questionnaires filled out by the drivers themselves. However, self-reported values can be subjective and biased [4,11,12] and are lower than actual values [2,3]. In this study, data rows were labeled in phase-2 according to a set of criteria developed with the help of traffic safety experts, thus making the profiling process more authentic. In addition, previous researchers analyzed driving parameters separately when profiling drivers. However, during the labeling process in phase-2, the effect of the correlation between those parameters on the profiling was considered.

Previous studies, such as those reported in related work, labeled the drivers as entirely safe or aggressive. Such labeling is considered impractical and unrealistic, according to experts, because drivers’ behavior constantly changes. In this study, the proposed profiling procedure lays out guidelines on how to provide continuous updates on drivers’ behavior. In [73], researchers merged the data into one file and measured the classification models’ performance on 1 to 5 min segments’ length. However, according to experts, even one minute is considered a lot, as behaviors leading to accidents may change within seconds. Therefore, data were merged into a single file in phase-1 and then divided into various timeframe segments in phase-3, which resulted in the model providing continuous updates on drivers’ changing behavior while enhancing its accuracy.

It is important to note that row labeling in phase-2 and segment labeling in phase-3 led to the construction of the driver profile scale, which is a scale that defines when specific behaviors are considered aggressive or safe according to the traffic laws and regulations in Malaysia. Such a scale could serve as a blueprint for future researchers interested in this domain.

Few studies in the literature proposed a deep-learning-based driver profiling recognition system. Those studies provided no recommendations for improving the accuracy of their models. Phase-4 involves normalizing data before feeding it into the models to avoid poor modeling due to features having different values on different scales. In phase-5, the dataset was divided into three sets: training, validation, and testing. The train-validate-test process in phase-6 provided consistent and reliable feedback on whether the models were overfitting to the data on the training set. In addition, the Earlystopping function was used to prevent the algorithms from running too long and causing overfitting during training. Moreover, the Dropout regularization technique was used to avoid overfitting and force the neural network to learn more robust features that are useful in conjunction with many different random subsets of the other neurons. The number of epochs and batch size were fine-tuned through trial and error to achieve the optimum performance for the models. An activation function, such as Relu, was used because it is computationally efficient and provides better results than other functions. Softmax was employed for the output layer.

Binary Cross-Entropy was used to evaluate the algorithms in modeling the data. Optimizers such as Adam and Nadam were used to adjust the models based on the output of the loss functions. Nadam achieved better accuracy for training the DNN model, while Adam achieved better accuracy for training the RNN and CNN models. Phases 4, 5, and 6 serve as guidelines to future researchers on modulating various deep-learning algorithms, such as DNN, RNN, and CNN, to obtain optimum performance while preventing overfitting issues during training.

The performance of the deep-learning algorithms was compared across various timeframe segments ranging from 1 to 10 s. Even though CNN is primarily used nowadays for image classification, it produced the most accurate results when compared to DNN and RNN. To the author’s knowledge, there is a gap in the current literature on the performance of deep-learning algorithms in the driver profiling domain, as most existing studies utilize machine-learning algorithms in proposing their recognition systems. The study’s results lead to the conclusion that CNN is better suited for the proposed recognition system than the other two algorithms. Such findings should encourage future researchers to utilize CNN for purposes other than image recognition.

Currently, accidents are increasing despite the various preventive measures put in place by the government, such as on-road cameras and warning signs. Such methods are ineffective due to the lack of randomization in the selection of observational sites. The data extracted from the camera is inevitably imprecise, therefore, reducing the reliability of those methods. In addition, police crash reports are usually written after accidents occur, and post-crash analysis is sometimes biased and prone to human errors. The data acquisition system and the proposed recognition system can help detect errant driving behavior for insurance companies. In addition, police can now use such a system to understand how and why accidents happen in Malaysia by extracting pre-crash data analysis from the system. The proposed recognition system can be used as an online application that reliably monitors driver behavior. Furthermore, when drivers are aware that their actions are being tracked, they tend to drive more cautiously, which helps lower accident rates. The main contributions of this study are:

1. Providing a profiling procedure that identifies safe and aggressive driving in Malaysia. As far as the author’s knowledge, this is the first established profiling procedure tailored for drivers in Malaysia and is aligned with traffic laws and regulations of the country.
2. Proposing a recognition system that can detect errant driving based on the established profiling procedure. This study proposes three modulated deep-learning models to choose for the recognition system. Experts’ guidelines were followed throughout the data labeling process. Several techniques were used to modulate the models, which could serve as guidelines for future research. Moreover, the study compares the performance of various deep-learning models and selects the best one for the recognition system, which is something that previous studies in this domain have yet to consider.
3. The proposed recognition system can be used by insurance firms to track drivers’ aggressive conduct on seconds basis. Furthermore, by pulling pre-crash data from the system, traffic officers would be able to obtain a better understanding of how and why accidents occur and what preventive steps should be taken in the future.

## 8. Conclusions

This research builds on a prior study in which the authors developed an acquisition system and gathered driving data from 30 participants. This is the first study to establish profiles for distinguishing aggressive and safe behaviors in Malaysia, in compliance with applicable traffic laws and regulations. A driver behavior profiling model based on timeframe data segmentation was proposed in this study. Safety score criteria for labeling data rows and timeframe segments in accordance with current traffic laws and regulations were outlined. Driving data were processed, segmented, and labeled. Then, three deep-learning-based algorithms, namely DNN, RNN, and CNN, were trained to classify the segments according to the established labels. Various techniques were employed to enhance the algorithms’ classification accuracy. Hyperparameters such as epochs, batch size, learning rate, and optimizers were fine-tuned, and functions such as Earlystopping and Dropout were utilized to prevent overfitting issues during the training process. Validation results showed good-fit modeling for the classification algorithms. The classification algorithms were tested on segments of 1 to 10 s in length. A confusion matrix was deployed to test the performance of the classification algorithms. Results showed that CNN performed much better than DNN and RNN across all timeframes, and was suggested for a recognition system that tracks and detects dangerous driving behaviors in Malaysia. The government and insurance firms could use the system to grant insurance exemptions as incentives for safe drivers while imposing more severe penalties on high-risk drivers, such as increased insurance rates. Additionally, by extracting pre-crash data from the system, traffic police would be able to gain a better understanding of how and why accidents occur, as the government’s current preventive measures are failing to reduce accident rates. In contrast with prior studies in which the driver receives a safety score, in this study, the dataset was divided into segments of 1–10 s in length, with each segment obtaining a safety score of 0 or 1 (0 indicates safe behavior, and 1 indicates aggressive behavior). This is a more rational and practical approach because drivers’ behavior varies over time, and no driver is constantly safe or aggressive. Safe and aggressive conduct is a state that changes over time when driving, and this study demonstrated how to model and recognize those behavioral changes. The profiling procedure presented in this study should serve as a blueprint for future researchers interested in this topic.

In the future, the authors plan to compare the performance of additional deep-learning algorithms, such as LSTM (Long Short-Term Memory), to those used in this study. Moreover, the authors aim to develop an online recognition system that utilizes the CNN algorithm for the detection of errant driving in Malaysia.

## Figures and Tables

**Table 1:** Data collection procedures in Malaysia.

Year	Type of Data Collection	Reference
2014	Observations	[21,22,23]
2015	Questionnaire	[24,25]
2015	Simulations	[26]
2015	Observations	[27]
2016	Observations	[28,29,30]
2017	Non-naturalistic experiments	[31]
2018	Questionnaire	[32,33]
2018	Simulations	[34]
2019	Questionnaire	[35,36]
2020	Observations	[37]
2020	Questionnaire	[38]

**Table 2:** Previous methods used in recognition systems.

Reference	Year	Type of Model
[39]	2017	Proposed machine-learning-based model using Random Forest (RF).
[18]	2017	Proposed four models. Three algorithms are machine-learning-based (RF, K-Nearest Neighbor (KNN), Adaboost), one is deep-learning-based Artificial Neural Network (ANN), and then compared the performance of those algorithms.
[31]	2017	Proposed deep-learning-based model using ANN.
[12]	2017	Proposed deep-learning-based model using SOM, which is a type of ANN.
[44]	2020	Proposed five models. Four of them are machine-learning-based (Support Vector Machine (SVM), RF, Fuzzy Logic, KNN), one is deep-learning-based (ANN), and then compared their performances.

**Table 3:** Criteria for safe and aggressive behaviors.

Parameter	Criteria	Status
Speed	<speed limit	Safe
	>speed limit	Aggressive
Distance to Vehicle Ahead	>4 m for every 15 kmh	Safe
	<4 m for every 15 kmh	Aggressive
Acceleration	<3.5 m/s^2^	Safe
	>3.5 m/s^2^	Aggressive
Deceleration	>−5.5 m/s^2^	Safe
	<−5.5 m/s^2^	Aggressive
Steering	If z-score for the change in yaw axis per second is between 1σ and −1σ	Safe
	If z-score for the change in yaw axis per second is above 1σ or below −1σ	Aggressive

Z-score is a numerical measurement that describes a value’s relationship to the mean of a group of values. It is denoted as z = (x − μ)/ σ, where x is the change in the yaw axis per second; μ is the mean; and σ is the standard deviation.

**Table 4:** Demonstration of the row labeling process.

Row Number	Distance to Vehicle Ahead (cm)	Speed (km/h)	Acceleration (m/s^2^)	Deceleration (m/s^2^)	Changes in Yaw Steering per Second (deg/s)	Row Safety Score
#1	266	20	0.55	0	11.98	1
#2	1000	28	2.22	0	10.01	0
#3	2240	50	6.11	0	15.6	1
#4	2607	61	3.05	0	10.38	0
#5	2943	28	0	−9.16	5.12	1
#6	2810	51	6.38	0	9.11	1
#7	3265	60	2.5	0	8.91	0
#8	3150	90	8.33	0	6.17	1
#9	3331	65	0	−6.94	23.1	1
#10	3940	63	0	-0.55	8.66	0

**Table 5:** Performance metrics.

Metric	Definition	How to Measure
Accuracy	Is the ratio of correctly predicted observations to the total observations.	(TP + TN)/(Positives + Negatives)
Recall	Is the ratio of correctly predicted positive observations to all observations in the actual class.	TP/(TP + FN)
Precision	Is the ratio of correctly predicted positive observations to the total predicted positive observations.	TP/(TP+ FP)
F-Measure	The weighted average of precision and recall.	2 × (Recall × Precision)/(Recall + Precision)

Reproduced from Al-Hussein et al. [10].

**Table 6:** Performance of modulated algorithms on segments 1–10 s long.

Segment Length	Accuracy (Training)	Accuracy (Testing)	Precision (Testing)	Recall (Testing)	F-Measure (Testing)
1 s	DNN (96.3%)	DNN (93.6%)	DNN (92.2%)	DNN (94.4%)	DNN (93.1%)
	RNN (93.9%)	RNN (93.1%)	RNN (91.8%)	RNN (93.6%)	RNN (92.6%)
	CNN (97.0%)	CNN (94.3%)	CNN (93.3%)	CNN (94.3%)	CNN (93.8%)
2 s	DNN (95.4%)	DNN (93.3%)	DNN (90.3%)	DNN (94.0%)	DNN (92.0%)
	RNN (92.2%)	RNN (92.8%)	RNN (90.7%)	RNN (90.9%)	RNN (90.8%)
	CNN (96.7%)	CNN (96.1%)	CNN (94.4%)	CNN (96.0%)	CNN (95.2%)
3 s	DNN (93.7%)	DNN (92.6%)	DNN (90.8%)	DNN (93.5%)	DNN (91.9%)
	RNN (91.1%)	RNN (91.1%)	RNN (89.3%)	RNN (91.2%)	RNN (90.1%)
	CNN (95.7%)	CNN (93.9%)	CNN (92.3%)	CNN (94.4%)	CNN (93.2%)
4 s	DNN (93.6%)	DNN (93.4%)	DNN (91.2%)	DNN (92.5%)	DNN (91.8%)
	RNN (91.2%)	RNN (92.8%)	RNN (90.4%)	RNN (91.8%)	RNN (91.1%)
	CNN (96.1%)	CNN (93.6%)	CNN (90.7%)	CNN (94.5%)	CNN (92.4%)
5 s	DNN (92.2%)	DNN (91.6%)	DNN (90.0%)	DNN (90.1%)	DNN (90.4%)
	RNN (90.8%)	RNN (91.2%)	RNN (89.5%)	RNN (90.3%)	RNN (89.9%)
	CNN (95.1%)	CNN (95.1%)	CNN (94.2%)	CNN (94.4%)	CNN (94.3%)
6 s	DNN (92.2%)	DNN (90.4%)	DNN (87.0%)	DNN (90.6%)	DNN (88.5%)
	RNN (91.0%)	RNN (90.9%)	RNN (88.9%)	RNN (88.1%)	RNN (88.4%)
	CNN (90.5%)	CNN (92.9%)	CNN (89.8%)	CNN (94.4%)	CNN (91.6%)
7 s	DNN (91.8%)	DNN (90.6%)	DNN (90.0%)	DNN (87.5%)	DNN (88.6%)
	RNN (89.0%)	RNN (90.1%)	RNN (89.0%)	RNN (87.5%)	RNN (88.2%)
	CNN (94.6%)	CNN (94.0%)	CNN (92.2%)	CNN (94.3%)	CNN (93.1%)
8 s	DNN (92.5%)	DNN (90.0%)	DNN (87.0%)	DNN (88.2%)	DNN (87.5%)
	RNN (89.7%)	RNN (90.0%)	RNN (87.0%)	RNN (88.3%)	RNN (87.6%)
	CNN (95.9%)	CNN (95.5%)	CNN (93.5%)	CNN (95.4%)	CNN (94.5%)
9 s	DNN (91.2%)	DNN (82.8%)	DNN (80.1%)	DNN (82.9%)	DNN (81.5%)
	RNN (90.3%)	RNN (84.7%)	RNN (81.7%)	RNN (82.7%)	RNN (82.1%)
	CNN (95.3%)	CNN (90.3%)	CNN (87.0%)	CNN (90.9%)	CNN (88.4%)
10 s	DNN (92.9%)	DNN (90.6%)	DNN (88.6%)	DNN (87.0%)	DNN (87.8%)
	RNN (90.2%)	RNN (91.9%)	RNN (90.1%)	RNN (88.9%)	RNN (90.0%)
	CNN (94.8%)	CNN (94.8%)	CNN (94.6%)	CNN (91.9%)	CNN (93.2%)

## Data Availability

Provided by the authors upon request.
